# Supplementary material for: A Critical View of the Application of the APEX Software (Aqueous Photochemistry of Environmentally-Occurring Xenobiotics) to Predict Photoreaction Kinetics in Surface Freshwaters
Source: Molecules. 2019 Dec 18;25(1):9. doi: 10.3390/molecules25010009 (PMC7017383; doi:10.3390/molecules25010009)
Supplement: Supplementary file 1 [file molecules-25-00009-s001.zip › 3.2.4_gcc-4.4.0.zip!/3.2.4_gcc-4.4.0/3.2.4_gcc-4.4.0/doc/octave/html/interpreter/Creating-Structures.html]

Creating Structures - Untitled


Next: Manipulating Structures,
Previous: Structure Arrays,
Up: Data Structures


---

#### 6.1.3 Creating Structures

As well as indexing a structure with ".", Octave can create a structure
with the `struct` command. `struct` takes pairs of arguments,
where the first argument in the pair is the fieldname to include in the
structure and the second is a scalar or cell array, representing the
values to include in the structure or structure array. For example

```
     struct ("field1", 1, "field2", 2)
     => ans =
           {
             field1 =  1
             field2 =  2
           }
```

If the values passed to `struct` are a mix of scalar and cell
arrays, then the scalar arguments are expanded to create a
structure array with a consistent dimension. For example

```
     s = struct ("field1", {1, "one"}, "field2", {2, "two"},
             "field3", 3);
     s.field1
          =>
             ans =  1
             ans = one
     
     s.field2
          =>
             ans =  2
             ans = two
     
     s.field3
          =>
             ans =  3
             ans =  3
```

If you want to create a struct which contains a cell array as an
individual field, you have to put it into another cell array like in
the following example:

```
     struct ("field1", {{1, "one"}}, "field2", 2)
          => ans =
             {
               field1 =
     
             {
               [1,1] =  1
               [1,2] = one
             }
     
               field2 =  2
             }
```

— Built-in Function: **struct** ("field", value, "field", value, ...)  
> Create a structure and initialize its value.
>
> If the values are cell arrays, create a structure array and initialize
> its values. The dimensions of each cell array of values must match.
> Singleton cells and non-cell values are repeated so that they fill
> the entire array. If the cells are empty, create an empty structure
> array with the specified field names.
>
> If the argument is an object, return the underlying struct.

The function `isstruct` can be used to test if an object is a
structure or a structure array.

— Built-in Function: **isstruct** (expr)  
> Return 1 if the value of the expression expr is a structure.
